# Red Light Control of β-Carotene Isomerisation to *9*-cis β-Carotene and Carotenoid Accumulation in *Dunaliella salina*

**DOI:** 10.3390/antiox8050148

**Published:** 2019-05-27

**Authors:** Yanan Xu, Patricia J. Harvey

**Affiliations:** Faculty of Engineering and Science, University of Greenwich, Central Avenue, Chatham Maritime, Kent ME4 4TB, UK; y.xu@greenwich.ac.uk

**Keywords:** *9-cis* β-carotene, *all-trans* β-carotene, *Dunaliella salina*, red LED, blue LED, growth, light intensity, carotenoids, isomerisation

## Abstract

*Dunaliella salina* is a rich source of *9-cis* β-carotene, which has been identified as an important biomolecule in the treatment of retinal dystrophies and other diseases. We previously showed that chlorophyll absorption of red light photons in *D. salina* is coupled with oxygen reduction and phytoene desaturation, and that it increases the pool size of β-carotene. Here, we show for the first time that growth under red light also controls the conversion of extant *all-trans* β-carotene to *9-cis* β-carotene by β-carotene isomerases. Cells illuminated with red light from a light emitting diode (LED) during cultivation contained a higher *9-cis* β-carotene content compared to cells illuminated with white or blue LED light. The *9-cis*/*all-trans* β-carotene ratio in red light treated cultures reached >2.5 within 48 h, and was independent of light intensity. Illumination using red light filters that eliminated blue wavelength light also increased the *9-cis*/*all-trans* β-carotene ratio. With norflurazon, a phytoene desaturase inhibitor which blocked downstream biosynthesis of β-carotene, extant *all-trans* β-carotene was converted to *9-cis* β-carotene during growth with red light and the *9-cis*/*all-trans* β-carotene ratio was ~2. With blue light under the same conditions, *9-cis* β-carotene was likely destroyed at a greater rate than *all-trans* β-carotene (*9-cis*/*all-trans* ratio 0.5). Red light perception by the red light photoreceptor, phytochrome, may increase the pool size of anti-oxidant, specifically *9-cis* β-carotene, both by upregulating phytoene synthase to increase the rate of biosynthesis of β-carotene and to reduce the rate of formation of reactive oxygen species (ROS), and by upregulating β-carotene isomerases to convert extant *all-trans* β-carotene to *9-cis* β-carotene.

## 1. Introduction

Carotenoids are synthesized by photosynthetic organisms for light-harvesting and for photo-protection of the pigment-protein light-harvesting complexes and photosynthetic reaction centres in the thylakoid membrane [1,2,3,4]. *Dunaliella salina*, a halotolerant chlorophyte, is one of the richest sources of natural carotenoids, and accumulates up to 10% of the dry biomass as β-carotene under conditions that are sub-optimal for growth, i.e., high light intensity, sub-optimal temperatures, nutrient limitation and high salt concentrations [5,6,7,8]. Two pools of β-carotene have been identified, which may be distinguished on the basis of geometric isomer configuration, *cis* or *trans* (*Z*/*E*), and enzyme complement. Thylakoid β-carotene consists principally of *all-trans* β-carotene (*all-trans* βC), and may be constitutively expressed; the ‘accumulated’ β-carotene, which is found in globules of lipid and proline-rich, β-carotene globule protein (the βC-plastoglobuli) in the inter-thylakoid spaces of the chloroplast, appears in high concentration of both *cis*/*trans* (*Z*/*E*) configurations, ratio ~1 [5,9,10,11].

The occurrence of such high concentrations of *9-cis* βC in *D. salina* is of great pharmaceutical interest. *9-cis* βC has a higher antioxidant activity than *all-trans* βC, and may also be more efficient than *all-trans* βC in vivo [12,13]. *9-cis* βC has been proposed in treatments for retinal dystrophies, chronic plaque psoriasis and atherosclerosis and as an anti-ageing therapy [14,15,16,17,18]. Importantly, a synthetic pure preparation of *9-cis* βC has recently been shown to inhibit photoreceptor degeneration of eye cups from mice with a retinoid cycle genetic defect [19].

However, the mechanism and regulation of the biosynthesis of *9-cis* βC in *D. salina* is unclear. Using different inhibitors of β-carotene biosynthesis, Shaish et al. [20] found that all the intermediates between phytoene and β-carotene in cultures maintained under low light intensity and N-starvation contained similar ratios of *9-cis*/*all-trans* stereoisomers. They concluded that the isomerisation step must occur at or before phytoene, and that no further isomerisation was likely to occur during the further transformation of phytoene to β-carotene. On the other hand, in cultures maintained under light stress, *9-cis*/*all-trans* βC isomerases were identified in high concentrations in plastidic globules, and were shown in vitro to catalyse conversion of *all-trans* βC to *9-cis* βC, whilst the expression of the corresponding genes was enhanced under stress conditions [21]. The *9-cis*/*all-trans* βC ratio has been shown to increase four-fold and the β-carotene content two-fold when the culture temperature decreased from 30 ℃ to 10 ℃ [22], and to increase with increased light intensity [21,23,24], but to be independent of light wavelength within the photosynthetically active range [7]. There have also been reports of a higher *9-cis*/*all-trans* βC ratio in *D. salina* cultivated under low light intensities [25,26].

Recently, we showed that growth of *D. salina* under high intensity red light was associated with carotenoid accumulation and a high rate of oxygen uptake [1]. We proposed a mechanism for carotenoid synthesis under red light, which involved absorption of red light photons by chlorophyll to reduce plastoquinone in photosystem II, coupled with phytoene desaturation by a plastoquinol:oxygen oxidoreductase, with oxygen as electron acceptor. Partitioning of electrons between photosynthesis and carotenoid biosynthesis would depend on both red photon flux intensity and phytoene synthase upregulation by the red light photoreceptor, phytochrome.

In this paper, the effects of red, white and blue light on the β-carotene isomeric composition in *D. salina* were investigated. Isomerisation between *all-trans* and *9-cis* βC in *D. salina* was regulated by light wavelength but not light intensity, with red light shifting the equilibrium in the direction of *9-cis* βC production. In blue light, *9-cis* βC was more rapidly destroyed than *all-trans* βC.

## 2. Materials and Methods

### 2.1. Strains and Cultivation

**Figure 1:**
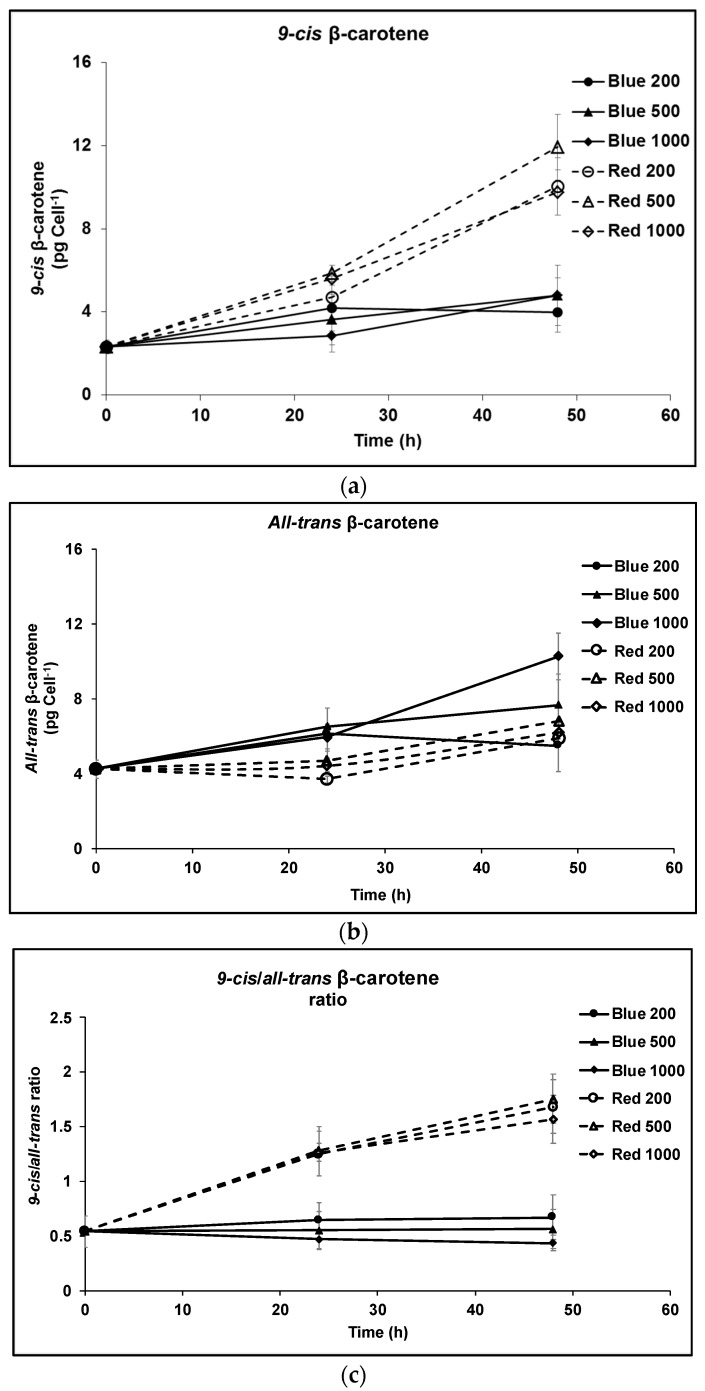

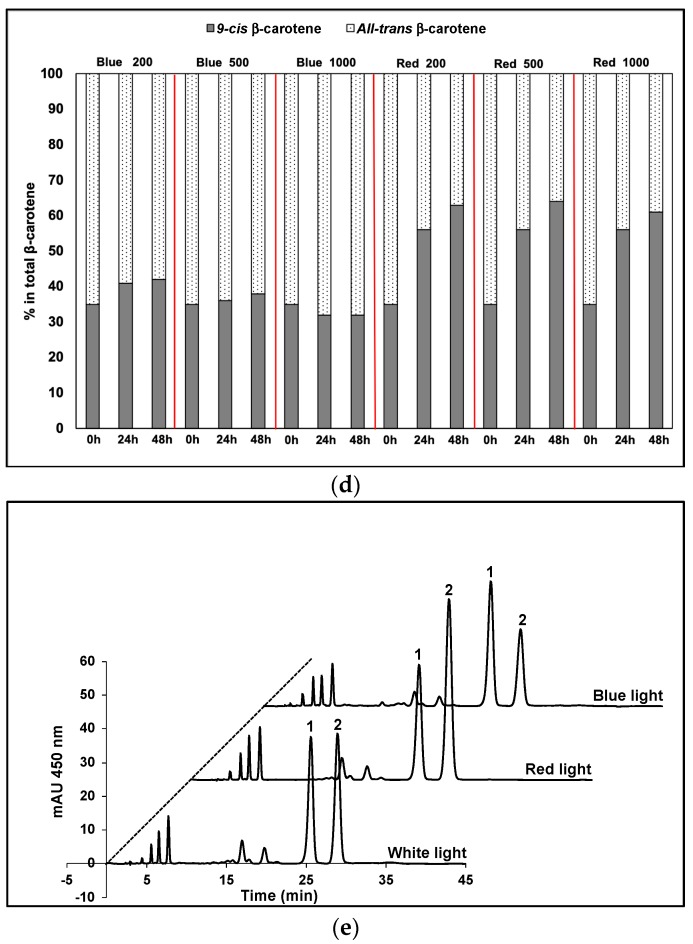
Cultivation of *D. salina* under continuous blue or red LED light at three different light intensities of 200, 500 and 1000 µmol m^−2^ s^−1^ for 48 h. (**a**) Cellular content of *9-cis* βC, (**b**) cellular content of *all-trans* βC; (**c**) *9-cis*/*all-trans* βC ratio. (**d**) Percentage of *9-cis* and *all-trans* βC in total βC. (**e**) HPLC profiles at 450 nm of carotenoid extracts from *D. salina* cultivated under continuous white light, red light or blue light, each at 1000 µmol m^−2^ s^−1^ for 48 h. Peak 1: *all-trans* β-carotene; peak 2: *9-cis* β-carotene. Biomass was collected at 48 h illumination and carotenoids extracted for HPLC analysis. Each culture condition was set up at least in triplicate. mAU: milli-absorbance unit.

**Figure A1:**
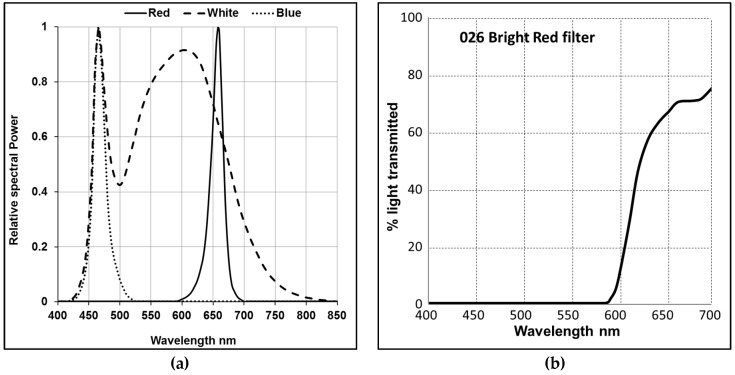

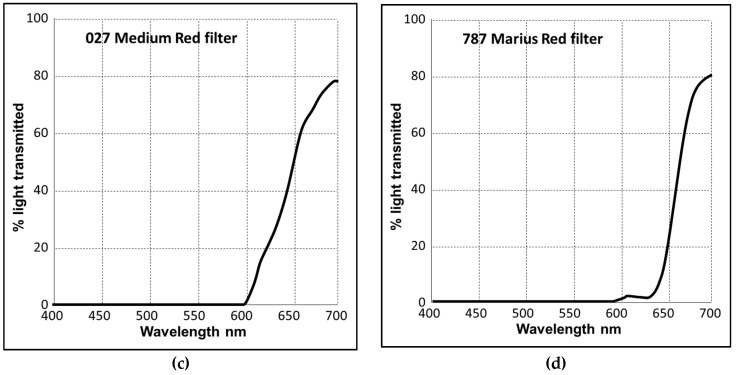
(**a**) Typical relative spectral power distribution of white, blue and red LED lights in the Algem bioreactor; (**b**–**d**) The light transmission (Y%) for each wavelength (nm) of filters that were used to transmit red light. (**b**) Lee Filters 026 Bright red (Transmission 8.6%), (**c**) 027 Medium Red (Transmission 3.6%), (**d**) 787 Marius Red (Transmission 1.0%).

*D. salina* strain CCAP 19/41 (PLY DF15) was isolated from a salt pond in Israel and obtained from the Marine Biological Association (MBA, Plymouth, UK). Algae were cultured in Modified Johnsons Medium [27] in an ALGEM Environmental Modeling Labscale Photobioreactor (Algenuity, Bedfordshire, UK) and growth was monitored as described previously [1]. For initial experiments described by Figure 1, *D. salina* cells were grown under 12/12 light/dark (L/D) with 200 µmol photons m^−2^ s^−1^ supplied by white light emitting diode (LED) light (Figure A1a) to exponential growth phase, then dark-adapted for 36 h. After dark adaptation, they were transferred to continuous white, blue or red LED light at light intensities of 200, 500, or 1000 μmol photons m^−2^ s^−1^ for 48 h. Samples were taken at 0, 24 and 48 h for carotenoids analysis. For experiments with norflurazon described by Figure 5, cultures were grown for 24 h under white LED light then norflurazon as added to cultures to a working concentration of 5 µM and maintained for a further 48 h under red, blue or a mix of red and blue LED light at 200 µmol m^−2^ s^−1^ or kept in the dark. Red filters (Lee filter 26 Bright red, 27 Medium red, and 787 Marius red (Figure A1b–d)) when used, were purchased from Lee Filters Andover (Hampshire, UK) and placed over the LED lights. The cultures were shaken for 10 min at 100 rpm every hour before taking samples to monitor cell growth in order to minimise sheer stress to the cells which have no cell wall.

### 2.2. Carotenoids Analysis

The composition of pigments was analysed by High-Performance Liquid Chromatography with Diode-Array Detection (HPLC-DAD) (Agilent Technologies 1200 series, Agilent, Santa Clara, United States). Biomass was harvested and extracted for HPLC analysis as described previously [1], and analysed at least in triplicate. A carotene standard for *all-trans* βC was obtained from Sigma-Aldrich Inc. (Merck KGaA, Darmstadt, Germany); a carotene standard for *9-cis* βC was obtained from Dynamic Extractions (Tredegar, Gwent, UK). The *all-trans* and *9-cis* βC contents were quantified from their absorption at 450 nm.

### 2.3. Statistical Analysis

Each experiment was carried out at least in triplicate. The collected data were analyzed in R by one way analysis of variance (ANOVA) with posterior Dunnett’s test and Turkey multiple pairwise-comparisons. A *p* < 0.05 value was considered significant.

## 3. Results

9-*cis* βC and *all-trans* βC were the major carotenoids that accumulated in *D. salina* biomass after 48 h exposure to red or blue LED light, but the relative pool sizes of each depended on the concentration of red and blue photons of light received. Under blue light, the contents of both *9-cis*- and *all-trans* βC per cell increased with time (Figure 1a,b), and the ratio of *cis*/*trans* βC isomers remained approximately the same at all light intensities (Figure 1c). The concentration of *9-cis* βC was ~half as much as *all-trans* βC. Under red light, by contrast, the concentration of *9-cis* βC and total pool of carotenoids increased massively compared to that in blue in all light intensities and the content of *9-cis* βC was ~twice as much as *all-trans* βC (Figure 1a,b). With increasing light intensity, the relative pool sizes of the isomers changed; that of *all-trans* βC decreased and that of *9-cis* βC increased. Furthermore *9-cis* βC increased with time to >60% of total β-carotene under red light (Figure 1d). HPLC profiles of the carotenoid extracts showed 9-*cis* βC and *all-trans* βC were the major carotenoids that accumulated in *D. salina* biomass, and that the ratios of the two isomers were different under different wavelengths (Figure 1e).

**Figure 2:**
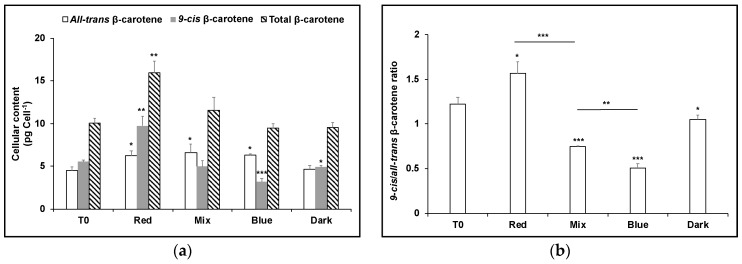
(**a**) Cellular content of *9-cis* βC and *all-trans* βC and (**b**) *9-cis*/*all-trans* βC ratio in *D. salina* cultures exposed to continuous red LED light at 1000 µmol m^−2^ s^−1^ for 24 h followed by 24 h under either red light, a mix of 1:1 red and blue light, blue light at the same light intensity of 1000 µmol m^−2^ s^−1^ or dark. Each culture condition was set up at least in triplicate. Results were analysed by one way analysis of variance (ANOVA) with posterior Dunnett’s test compared to T0 and Tukey multiple pairwise-comparisons. Asterisks represent different levels of significance (*** 0 < *p* ≤ 0.001, ** 0.001 < *p* ≤ 0.01, * 0.01 < *p* ≤ 0.05).

**Figure 3:**
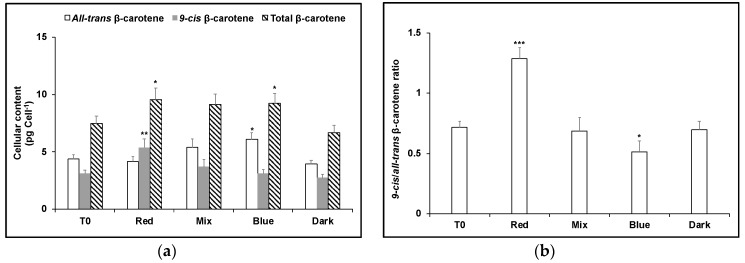
(**a**) Cellular content of *9-cis* βC and *all-trans* βC and (**b**) *9-cis*/*all-trans* βC ratio in *D. salina* cultures exposed to continuous blue LED light at 1000 µmol m^−2^ s^−1^ for 24 h followed by 24 h under either red light, a mix of 1:1 red and blue light, blue light at the same light intensity of 1000 µmol m^−2^ s^−1^ or dark. Each culture condition was set up at least in triplicate. Results were analysed by one way ANOVA with posterior Dunnett’s test compared to T0 and Tukey multiple pairwise-comparisons. Asterisks represent different levels of significance (*** 0 < *p* ≤ 0.001, ** 0.001 < *p* ≤ 0.01, * 0.01 < *p* ≤ 0.05).

To test the effect of blue light exposure on carotene isomers that had accumulated in red light and vice versa, dark-adapted cultures of *D. salina* were cultivated in red or blue LED high intensity light for 24 h (T0), and then cultivated for a further 24 h in red, blue, or a mixture of red and blue LED light (1:1) with the same light intensity, or the dark. As before, red-shifted cells maintained in red light produced the greatest amount of carotenoids with ~twice as much as *9-cis* βC as *all-trans* βC (Figure 2). On the other hand, *9-cis* βC decreased when red-shifted cells were transferred to blue light (Figure 2), to the same level as for blue-shifted cells maintained continuously in blue (Figure 3); the pool size of carotenoids for both conditions was about the same and the concentration of *9-cis* βC was ~half as much as *all-trans* βC. Conversely, blue-shifted cells when transferred to red LED produced more carotenoids (28% greater content), principally as *9-cis* βC (Figure 3).

**Figure 4:**
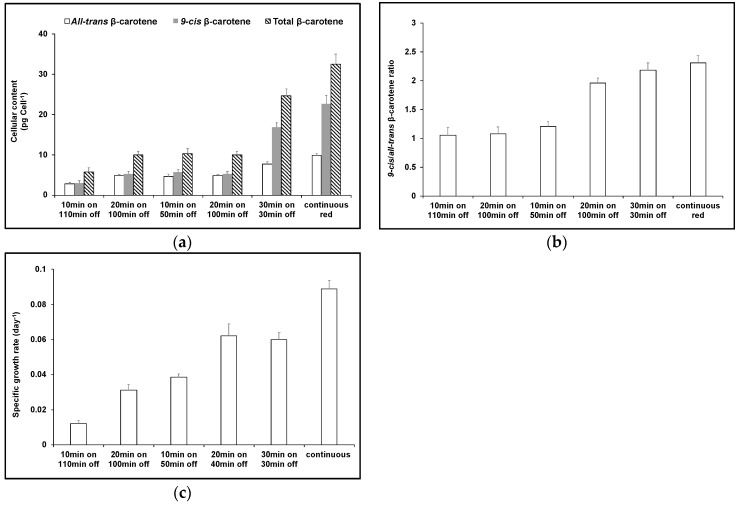
Effect of cultivating D. salina under different red light/dark cycles. (**a**) Cellular content of *9-cis* βC, *all-trans* βC and total βC and (**b**) *9-cis*/*all-trans* βC ratio and (**c**) specific growth rate of *D. salina* cultures grown under different light/dark cycles of red LED light supplied at 500 µmol m^−2^ s^−1^. Cultures of *D. salina* were grown to a cell density of ~0.2 million cells mL^−1^ under white LED light and then transferred into red LED light growth cycles of different duration. Carotenoids were analysed after 6 days growth.

Since red light increased the net content of *9-cis* βC, the effects of red light/dark cycles of increasing red light duration during cultivation were tested. Increasing red light duration increased the total amount of β-carotene, in particular the amount of *9-cis* βC (Figure 4). With a red light/dark cycle of 10 min/110 min, the ratio of *9-cis*/*all-trans* βC was 1.1, but in a red light/dark cycle of 30 min/30 min, this increased to 2.2, similar to that in continuous red (2.3). However, in continuous red light, the total pool size β-carotene was nearly 25% greater.

**Figure 5:**
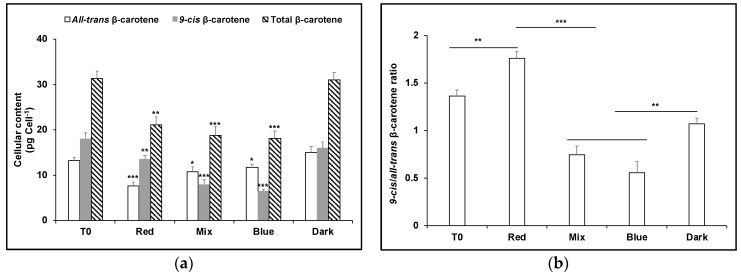
Production of carotenes in *D. salina* cultures treated with 5 µM norflurazon. (**a**) cellular content of *9-cis* βC, *all-trans* βC and total βC and (**b**) *9-cis*/*all-trans* βC ratio. Cultures were grown for 24 h under white LED light then treated with norflurazon and maintained for a further 48 h under red, blue or a mix of red and blue LED light at 200 µmol photons m^−2^ s^−1^ or kept in the dark. T0: time point after growth for 24h under white LED light only, before addition of norflurazon. Results were analysed by one way ANOVA with posterior Dunnett’s test compared to T0 and Tukey multiple pairwise-comparisons. Asterisks represent different levels of significance (*** 0 < *p* ≤ 0.001, ** 0.001 < *p* ≤ 0.01, * 0.01 < *p* ≤ 0.05).

The accumulation of carotenoids under red light has previously been shown to involve upregulation of phytoene synthase to increase the pool size of phytoene in *D. salina* cultures [1]. In order to test the effect of blue and red light on the β-carotene isomer composition, but without interference of de novo synthesis of β-carotene from phytoene, norflurazon, a phytoene desaturase inhibitor, was applied to the *D. salina* cultures (Figure 5). After 48 h without light, the total pool size of carotenoids was the same as that at the outset of the experiment (T0) before light treatment i.e., norflurazon blocked any further downstream synthesis of β-carotene. Under these conditions, the β-carotene isomer composition, *9-cis*/*all-trans* βC, was 1.1, the same as that recorded for growth in a red light/dark cycle of 10 min/110 min. Both red and blue light treatments lowered the total pool size of total β-carotene, blue more than red: ~31–32% total β-carotene was destroyed under red light and under the 1:1 red/ blue light mix, and ~41% under blue light. Carotenoids absorb photons in the range 400–550 nm, exactly overlapping the emission spectrum of the blue LED (440–500 nm) therefore the greater loss in blue light compared to red was to be anticipated. Furthermore, although both *all-trans* βC and *9-cis* βC were destroyed under blue light, the loss of *9-cis* βC was very much greater: only ~40% of the content of *9-cis* βC recorded in dark-treated cultures remained, compared to 78% for *all-trans* βC. Since *9-cis* βC has a higher antioxidant activity than *all-trans* βC, this result might also be anticipated. Somewhat surprisingly, however, loss of *9-cis* βC under red light compared to blue was much smaller and the ratio of *9-cis*/*all-trans* βC was 3-fold greater than under blue light. Since the emission spectrum of the red LED (625–680 nm) emits photons that are not absorbed by β-carotene, these data imply isomerisation of extant *all-trans* βC to *9-cis* βC to increase the content of *9-cis* βC at the expense of *all-trans* βC during growth.

**Figure 6:**
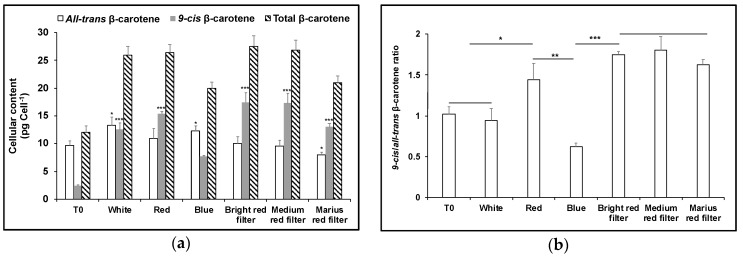
Cultivation of *D. salina* using red light filters. *D. salina* was cultivated under white light to early orange phase (cell density of ~0.5 × 10^6^ cells mL^−1^; carotenoid: chlorophyll ratio ~3), and then cultures were diluted with fresh medium to a cell density of ~0.2 × 10^6^ cells mL^−1^ (no nutrient stress) (T0) and then further cultivated for 48 h under white, red or blue LED light at 1000 µmol m^−2^ s^−1^ or under white LED light at 1000 µmol m^−2^ s^−1^ covered with one of three different red filters (Lee filter 26 Bright red; Lee filter 27 Medium red; or Lee filter 787 Marius red). (**a**) Cellular content of *9-cis*, *all-trans* and total β-carotene. (**b**) *9-cis*/*all-trans* β-carotene ratio. Results were analysed by one way ANOVA and Tukey multiple pairwise-comparisons. Asterisks represent different levels of significance (*** 0 < *p* ≤ 0.001, ** 0.001 < *p* ≤ 0.01, * 0.01 < *p* ≤ 0.05).

A similarly greater loss of *all-trans* βC compared to *9-cis* βC in red light was obtained using Lee Bright Red, Medium Red or 787 Marius Red filters: these transmitted only a fraction (8.6%, 3.6% and 1.0%) of the light intensity applied with a red LED (1000 µmol m^−2^ s^−1^), but importantly excluded light wavelengths below 550 nm (Figure A1b–d). Each increased the total β-carotene pool size and the *9-cis*/*all-trans* βC ratio was higher (Figure 6). With the 787 Marius Red filter, cells received only approximately 10–17 µmol m^−2^ s^−1^ light intensity of the red wavelength but this was still sufficient to increase the ratio of *9-cis*/*all-trans* βC ratio, the amount of *9-cis* βC per cell and total β-carotene to values approaching those found using white light at 1000 µmol m^−2^ s^−1^.

**Figure 7:**
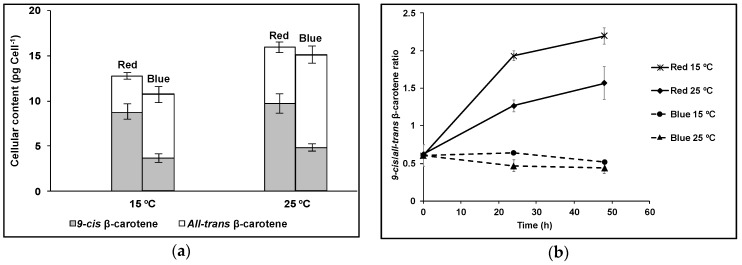
*D. salina* cultivated under red or blue light at either 15 ℃ or 25 ℃. (**a**) Cellular content of *9-cis* βC and *all-trans* βC (**b**) *9-cis*/*all-trans* βC ratio. Cells were cultured under a light:dark 12h:12h white light growth regime to mid-log phase of the growth cycle (0.1–0.2 × 10^6^ cells mL^−1^) then transferred to the dark for 24 h before treatment for 48 h at either 15 °C or 25 °C under continuous blue or red LED light at 1000 µmol m^−2^ s^−1^. Each culture condition was set up at least in triplicate.

The co-regulation by light and temperature on the β-carotene production and isomeric composition in *D. salina* is shown in Figure 7. Cultivation at 15 °C compared to 25 °C increased the *9-cis*/*all-trans* βC ratio, especially under red light, but decreased the pool size of β-carotene measured over the same time frame (48 h).

**Figure 8:**
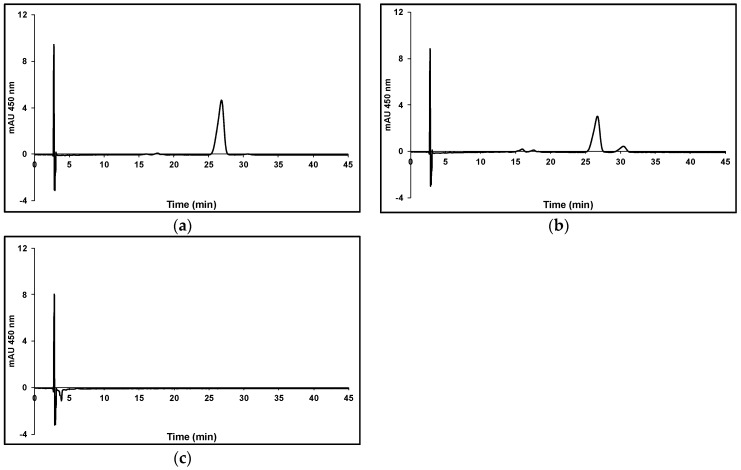
Effect of red or blue LED light on the photo-destruction of *all-trans* βC. *All-trans* βC was dissolved in chloroform to a final concentration of 2.4 μM and vials were thoroughly flushed with either nitrogen or air, sealed and incubated for 24 h at 25 °C under different LED lights at 200 µmol m^−2^ s^−1^. (**a**) Red light under nitrogen; (**b**) Red light in air; (**c**) Blue light in air.

Finally, the effects of blue and red light on the destruction of *all-trans* βC were evaluated. No reaction of *all-trans* βC solutions was detected under red light in nitrogen (Figure 8a). Under red light in air, (Figure 8b), 40% destruction of *all-trans* βC was recorded, whereas in blue light (Figure 8c), *all-trans* βC was fully destroyed within the same time frame. These data show that blue light is more damaging to *all-trans* βC than red light.

## 4. Discussion

In the present work, we found that under high intensity red LED light (up to 1000 µmol m^−2^ s^−1^) but in conditions of nutrient sufficiency, *D. salina* accumulated carotenoids rapidly within 48 h. Surprisingly, the major accumulated isomer was *9-cis* βC, ~twice as much as *all-trans* βC. In vitro, *9-cis* βC is a better scavenger of free radicals than *all-trans* βC [12], and reportedly degrades more rapidly compared to *all-trans* βC under both light and dark conditions [28]. Furthermore, chlorophyll absorbs photons in the range of the emission spectrum of the red LED used here (625–680 nm) and therefore in *D. salina* cultures in high intensity red light, a high rate of photo-oxidation of *9-cis* βC might have been anticipated. Carotenoids are known antioxidants synthesized by many microalgae to prevent photoinhibition caused by photo-oxidation of photosynthetic reaction centres. Photooxidative damage occurs when species such as singlet oxygen (^1^O_2_) are formed under saturating light conditions as a result of transfer of energy from chlorophyll in the triplet excited state (^3^Chl*) to the ground state of O_2_. ^1^O_2_ react readily with fatty acids to form lipid peroxides and will set up a chain of oxygen activation events that may eventually lead to a hyperoxidant state and cell death [29]. Carotenoids protect the photosystems in the following ways: (i) by reacting with lipid peroxidation products and terminating free radical chain reactions as a result of the presence of the polyene chain; (ii) by scavenging ^1^O_2_ and dissipating the energy as heat; and (iii) by reacting with triplet excited chlorophyll ^3^Chl* to prevent formation of ^1^O_2_ or by dissipation of excess excitation energy through the xanthophyll cycle [3,30,31].

The simplest explanation to resolve the seeming anomaly, namely accumulation of the more readily degraded *9-cis* βC under high intensity red light conditions that should be associated with high rates of photo-oxidation, invokes the activity of β-carotene isomerases, the gene transcripts of which are increased in light stress [21]. Davidi et al. [11] showed that all the enzymes in the biosynthetic pathway from phytoene to β-carotene were present in the plastidic lipid globules and included enriched concentrations β-carotene isomerases; two of these, *9-cis*-βC-ISO*1* and *9-cis*-βC-ISO*2*, were shown to be responsible for the catalytic conversion of *all-trans* βC to *9-cis* βC. Based on the data presented here we propose that the expression of gene transcripts of β-carotene isomerases may be triggered by specific light sensing, possibly through phytochrome.

In red light compared to blue, the apparent loss of *9-cis* βC with norflurazon was surprisingly small and the ratio of *9-cis*/*all-trans* βC was 3-fold greater than in blue light (Figure 5). Accumulation of *9-cis* βC by phytoene synthase (PSY) gene activation, whose expression has been shown to be greatly increased 6–48 h following stress [11] was precluded by the presence of norflurazon, which blocked phytoene desaturation and consequent carotene synthesis. Under these conditions, the relative increase in pool size of *9-cis* βC in red light implies a much higher rate of *9-cis* βC formation from extant *all-trans* βC, caused by increased isomerase activity, than the rate of *9-cis* βC destruction (see Figure 5). Carotenes absorb photons in the range 400–550 nm, exactly overlapping the emission spectrum of the blue LED (440–500 nm). However blue light catalysed a much more rapid rate of destruction of carotenes than red light (Figure 8). In blue LED light, *9-cis* βC would be destroyed more rapidly than could be replenished by adjustment of the *9-cis*/*all-trans* βC equilibrium position because increased β-carotene isomerase activity from red-light activated gene expression for β-carotene isomerases is not possible in blue light (see Figure 5).

Red light stimulation of the expression of gene transcripts of β-carotene isomerases by a phytochrome to increase the rate of accumulation of *9-cis* βC by β-carotene isomerases is also supported by the increase in pool size of *9-cis* βC under low intensity red light (Figure 6). Each of the Lee red light filters increased the total β-carotene pool size and the *9-cis*/*all-trans* βC ratio was higher despite the much lower light intensity of the red wavelength compared to the red LED light. The effects of low temperature on *9-cis* βC-accumulation in *D. salina* are also noteworthy, since enzyme catalysis typically shows a Q_10_ (temperature coefficient) ~2, yet in the present work, formation of *9-cis* βC in low temperature compared to high was increased under red light, and had little effect in blue. In higher plants, the activated phytochrome B, a red light photoreceptor, is considered to function as the thermal sensor to sense environmental temperature [32]. Mutants with no phytochromes showed a constitutive warm temperature transcriptome even at low temperatures [33]. Red light sensing to increase the concentration of β-carotene isomerases and catalyse conversion of *all-trans* βC at low temperatures, as well as high, may play a significant role in photoprotection in *D. salina*.

**Scheme 1:**
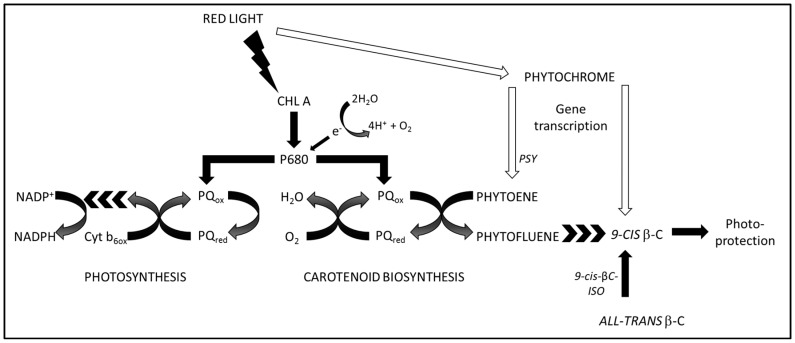
Regulation of the pool size of *9-cis* βC. Red photon flux intensity controls the partitioning of electrons either for carotenoid biosynthesis or for photosynthesis, via energy absorption by chlorophyll and the PQ pool [1]. Red photon flux also controls phytochrome regulation of the production of gene transcripts for phytoene synthase and β-carotene isomerases. CHL A: chlorophyll a; P680: chlorophyll a, primary electron donor of Photosystem II; PQ_ox_: plastoquinone, oxidised form; PQ_red_: plastoquinone, reduced form; Cyt b_6ox_: cytochrome b6f complex, oxidised form; NADP^+^: NADP oxidised form; NADPH: NADP reduced form; PSY: phytoene synthase; 9-*cis*-βC-ISO: *9-cis* βC isomerase.

We recently proposed that red light enhanced the production of carotenoids in a mechanism dependent on both photon flux density as well as upregulation of phytoene synthase by the red light photoreceptor phytochrome and that chlorophyll absorption of red light photons and subsequent plastoquinone reduction in photosystem II was coupled with oxygen reduction and phytoene desaturation by plastoquinol:oxygen oxidoreductase [1]. According to the findings in the previous work [1], the partitioning electron flux between photosynthesis and carotenoid biosynthesis could be augmented by addition of the regulation of the pool size of *9-cis* βC, as seen in the Scheme 1.

Red light sensing by phytochrome to increase the pool size of phytoene by phytoene synthase has been reported in higher plants [34]. Red light control of carotenoid biosynthesis coupled with the accumulation of the more readily oxidized *9-cis* βC as a consequence of isomerisation from *all-trans* βC reserves would therefore rapidly increase the pool size of anti-oxidant to reduce the rate of formation of ROS under stress (See Scheme 1).

## 5. Conclusions

Red light availability regulates the isomerisation of *all-trans* β-carotene to *9-cis* β-carotene and upregulates carotenoid biosynthesis in the halotolerant microalga *Dunaliella salina*. In red light *9-cis* βC accumulated, caused by increase in the rate of isomerisation of *all-trans* βC to *9-cis* βC relative to the rate of its destruction. Red light may have industrial value as an energy-efficient light source for production of natural *9-cis* βC from *D. salina*.
